# SGLT2 Inhibitors in Nephrolithiasis: Emerging Evidence and What Urologists Need to Know

**DOI:** 10.3390/medsci14050555

**Published:** 2026-09-09

**Authors:** Marius Ivănuță, Dragoș Puia, Ana-Maria Ivănuță, Alexandra Haută, Ionuț Nistor, Cătălin Pricop

**Affiliations:** 1 Department of Urology, Grigore T. Popa University of Medicine and Pharmacy Iasi, 700115 Iasi, Romania; marius.ivanuta@umfiasi.ro (M.I.); alexandra.hauta@umfiasi.ro (A.H.); ionut.nistor@umfiasi.ro (I.N.); catalin.pricop@umfiasi.ro (C.P.); 2 Department of Urology, “Dr. C.I. Parhon” Clinical Hospital, 700503 Iasi, Romania; 3 Center for Morphological and Spectroscopic Analysis of Urinary Stones” Michel Daudon”, 700503 Iasi, Romania; 4 Emergency Care Department, “Sf. Spiridon” County University Emergency Hospital, 700111 Iasi, Romania; anamariaion92@yahoo.com; 5 Department of Nephrology, “Dr. C.I. Parhon” Clinical Hospital, 700503 Iasi, Romania

**Keywords:** sodium–glucose cotransporter 2 inhibitors, nephrolithiasis, urolithiasis, pathophysiology, metabolic syndrome

## Abstract

Background: Nephrolithiasis frequently coexists with type 2 diabetes, obesity, gout, chronic kidney disease, and other cardiometabolic disorders. Sodium–glucose cotransporter 2 (SGLT2) inhibitors may influence stone formation through effects on glucosuria, natriuresis, urine volume, urinary citrate, pH, urate handling, and relative supersaturation. This review aimed to examine the current mechanistic, biochemical, and clinical evidence linking SGLT2 inhibition to nephrolithiasis, with particular attention to its relevance for urological practice. Methods: A clinically oriented narrative review was conducted using PubMed/MEDLINE, Scopus, Web of Science, and Google Scholar. The final search was performed on 1 July 2026. A total of 15 studies were included. Randomised trials, pooled and post hoc analyses, observational and comparative-effectiveness studies, meta-analyses, human mechanistic studies, and relevant experimental investigations were considered. Evidence was synthesised narratively according to biological mechanisms, urinary chemistry and relative supersaturation, randomised evidence, and observational clinical outcomes. Clinical stone events were analysed separately from surrogate urinary endpoints. Results: SGLT2 inhibition is associated with reproducible increases in urinary citrate and variable effects on urine volume, urinary pH, and the excretion of calcium, phosphate, and urate. Short-term mechanistic studies suggest reductions in calcium phosphate supersaturation and, in selected uric acid stone formers, uric acid supersaturation, whereas effects on calcium oxalate supersaturation remain inconsistent. Observational studies and secondary analyses of randomised trials generally indicate fewer nephrolithiasis events among SGLT2 inhibitor users; however, most outcomes were based on adverse-event reporting or administrative codes and lacked stone composition, standardised imaging, urinary phenotyping, and recurrence adjudication. Conclusions: SGLT2 inhibitors may favourably modify selected lithogenic pathways, but their effects appear phenotype-dependent and have not yet been shown to directly prevent stone recurrence. They should not be initiated solely for the prevention of nephrolithiasis. In patients with established cardiovascular, renal, or metabolic indications, their potential urinary effects may represent an additional benefit. Dedicated long-term randomised studies with stone-specific endpoints are required.

## 1. Introduction: Why Kidney Stone Prevention Needs Rethinking

### 1.1. The Unmet Need in Kidney Stone Prevention

Kidney stone disease remains common and often recurrent despite advances in diagnostic imaging and minimally invasive treatment. Acute episodes are usually managed effectively with observation, medical expulsive therapy, ureteroscopy, shock-wave lithotripsy, or percutaneous intervention. However, stone removal does not address the metabolic and environmental factors responsible for lithogenesis, and many patients subsequently develop new calculi, recurrent renal colic, obstruction, infection, or require further procedures [1,2].

Current preventive strategies include increased fluid intake, dietary modification, correction of urinary abnormalities, and selected pharmacological therapies. Their effectiveness may nevertheless be limited by adherence, adverse effects, incomplete metabolic response, and the coexistence of multiple risk factors.

Many stone formers also present with obesity, type 2 diabetes, hypertension, dyslipidaemia, gout, metabolic syndrome, or chronic kidney disease, supporting the concept that nephrolithiasis may occur within a broader cardiorenometabolic context. This has prompted interest in therapies that may influence both systemic metabolic disease and lithogenic risk. SGLT2 inhibitors are of particular interest because their established cardiorenal effects are accompanied by changes in several urinary parameters relevant to stone formation. Emerging clinical data also suggest fewer nephrolithiasis events among patients receiving these agents compared with those treated with alternative glucose-lowering therapies [3,4].

### 1.2. The Metabolic Overlap Between Nephrolithiasis and Cardiometabolic Disease

Nephrolithiasis is frequently associated with obesity, type 2 diabetes mellitus, hypertension, dyslipidaemia, gout, and chronic kidney disease.

Insulin resistance is particularly relevant to uric acid stone formation. In patients with obesity or type 2 diabetes, impaired proximal tubular ammonium production reduces urinary buffering capacity and promotes persistently acidic urine. Under these conditions, uric acid solubility decreases, and crystallisation may occur even without marked hyperuricosuria. Consistent with this mechanism, type 2 diabetes is strongly associated with uric acid stone composition and is an important metabolic predictor of this phenotype [5,6].

Obesity and metabolic syndrome may also increase the risk of calcium stone formation through dietary acid load, high sodium intake, hypocitraturia, altered calcium excretion, and reduced urine volume. Because these abnormalities often coexist, the resulting lithogenic phenotype may not be adequately addressed by targeting a single urinary parameter [6,7].

Gout and hyperuricaemia further demonstrate the close relationship between systemic metabolism and stone disease. Patients with gout have a higher risk of nephrolithiasis, and pure or mixed uric acid stones are common in this population [5]. Conversely, nephrolithiasis has been associated with a higher subsequent risk of chronic kidney disease and other cardiometabolic disorders, suggesting a bidirectional relationship rather than simple coexistence [1,3].

For the urologist, this overlap has direct clinical relevance. The evaluation of recurrent stone formers should include not only stone composition and urinary abnormalities but also the broader metabolic context. Diabetes, obesity, gout, and chronic kidney disease may influence stone phenotype, recurrence risk, preventive strategy, and the selection of concomitant medical therapy [8].

### 1.3. Why SGLT2 Inhibitors Are Relevant to Urologists

The relevance of SGLT2 inhibitors to urology has increased in parallel with their expanding use in patients with type 2 diabetes, chronic kidney disease, heart failure, and other cardiometabolic disorders. These conditions are highly prevalent among stone formers and are closely linked to metabolic phenotypes characterised by acidic urine, hyperuricemia, hypocitraturia, obesity, hypertension, and recurrent nephrolithiasis [3,4]. Consequently, urologists are increasingly likely to encounter patients with stone disease who are already receiving, or may soon be considered for, SGLT2 inhibitor therapy. Beyond their systemic indications, SGLT2 inhibitors may influence several urinary determinants of lithogenesis. By inducing glucosuria and altering proximal tubular transport, these agents can affect urine volume, sodium handling, uric acid excretion, urinary citrate, and acid–base balance [4,5]. These changes are clinically relevant because stone formation is governed not by a single urinary abnormality but by the combined effect of urine volume, pH, lithogenic solute excretion, crystallisation inhibitors, and relative supersaturation. Available mechanistic studies suggest potentially favourable effects on citrate excretion and selected supersaturation profiles, although the direction and clinical significance of these changes may vary according to stone composition and baseline urinary phenotype [6,7]. Clinical interest has also been driven by outcome data. Across several observational and comparative-effectiveness studies, SGLT2 inhibitor use has been associated with fewer nephrolithiasis events than other glucose-lowering strategies [1,2,3].

### 1.4. Aim and Scope of the Review

The potential relationship between SGLT2 inhibition and kidney stone disease has recently attracted attention, yet the available evidence remains heterogeneous. Data derive from large clinical studies of patients treated for cardiometabolic indications, observational analyses of nephrolithiasis outcomes, short-term studies of urinary biochemical changes, and experimental models addressing tubular injury and crystal retention. These lines of evidence suggest biological plausibility, but they have not yet been integrated into a clinically coherent framework for urological practice.

This review aims to examine the current evidence on SGLT2 inhibitors and nephrolithiasis from a urological perspective. Mechanistic and urinary biochemical findings are considered alongside clinical data on stone events, with an emphasis on their relevance to clinical practice. The practical implications are discussed in the context of the available evidence and should not be interpreted as formal treatment recommendations.

The review is deliberately translational in scope. It does not evaluate SGLT2 inhibitors as established stone-preventive therapy but instead examines whether their metabolic and renal effects may inform future strategies for nephrolithiasis prevention. By distinguishing mechanistic findings from clinical outcome data, this review seeks to clarify what is currently supported by evidence and what remains to be addressed in dedicated stone-focused studies.

## 2. Materials and Methods

### 2.1. Review Design

This study was conducted as a clinically oriented narrative review of the literature on sodium–glucose cotransporter 2 inhibitors and nephrolithiasis. The review aimed to integrate mechanistic, biochemical, experimental, and clinical evidence relevant to urological practice, with particular emphasis on urinary chemistry, relative supersaturation, stone phenotype, and clinically reported outcomes of nephrolithiasis. The review was not designed as a systematic review or meta-analysis; rather, it sought to provide a critical and clinically contextualised synthesis of the most relevant available evidence.

### 2.2. Information Sources and Search Strategy

A structured literature search was performed in PubMed/MEDLINE, Scopus, Web of Science, and Google Scholar for studies published between January 2000 and 1 July 2026. The final search in all databases was performed on 1 July 2026. Search terms combined SGLT2-related terms with nephrolithiasis and urinary risk parameters.

This review was registered with the International Prospective Register of Systematic Reviews (PROSPERO; registration number CRD420261453266).

### 2.3. Eligibility Criteria

Priority was given to randomised trials, pooled and post hoc analyses of randomised trials, observational cohort studies, comparative-effectiveness studies, meta-analyses, human mechanistic studies, and experimental investigations directly relevant to lithogenesis. Studies were considered eligible if they reported nephrolithiasis incidence or recurrence, stone-related procedures, changes in urinary biochemical parameters, relative supersaturation, or mechanisms involving crystal formation, tubular injury, oxidative stress, inflammation, autophagy, crystal adhesion, or crystal retention.

Studies were excluded if they did not evaluate SGLT2 inhibitors, did not report stone-related outcomes or mechanistically relevant findings, focused exclusively on glycaemic, cardiovascular, or renal endpoints, lacked sufficient methodological detail, or were not published in English. Editorials, letters, and expert opinions without original data were not used as primary evidence, although narrative reviews were consulted for background information and to identify references.

Titles and abstracts were screened for relevance, followed by full-text assessment of potentially eligible publications. Study selection was guided by the prespecified scope of the review and by the clinical or mechanistic relevance of each study to nephrolithiasis.

### 2.4. Study Selection and Data Extraction

Database searching identified 208 records: 72 from PubMed/MEDLINE, 61 from Scopus, 54 from Web of Science, and 21 from Google Scholar. Six additional records were identified through reference-list screening, resulting in a total of 214 records. After removal of 76 duplicates, 138 records underwent title and abstract screening. Of these, 101 were excluded because they were unrelated to nephrolithiasis or did not evaluate SGLT2 inhibitors, leaving 37 publications for full-text assessment. Twenty-two publications were excluded at this stage: nine did not report stone-related or mechanistically relevant outcomes, five focused exclusively on general glycaemic, cardiovascular, or renal outcomes, four were narrative reviews, editorials, or opinion articles without original data, two lacked sufficient methodological detail, and two reported overlapping data. The final evidence synthesis therefore included 15 studies.

For each included study, information was collected on study design, population, SGLT2 inhibitor, comparator, treatment duration, follow-up, nephrolithiasis-related endpoints, urinary biochemical parameters, relative supersaturation, principal findings, and relevant methodological limitations.

Because of substantial heterogeneity in study design, populations, interventions, follow-up duration, and outcome definitions, no quantitative pooling was performed. Evidence was synthesised narratively and organised into four principal domains: biological mechanisms, urinary chemistry and relative supersaturation, randomised and randomised-derived evidence, and observational clinical outcomes.

Clinical stone events were interpreted separately from surrogate urinary endpoints, with particular attention to distinctions between incident nephrolithiasis, recurrent stone events, diagnosis-code-based outcomes, stone-related procedures, and changes in stone-specific relative supersaturation.

No formal risk-of-bias assessment was undertaken, consistent with the narrative design. However, relevant study limitations, including outcome misclassification, residual confounding, absence of stone composition, lack of standardised imaging, and limited urinary phenotyping, were considered when interpreting the strength and clinical applicability of the evidence.

## 3. Why SGLT2 Inhibitors May Influence Stone Formation

### 3.1. Renal Tubular Effects of SGLT2 Inhibition

SGLT2 is predominantly expressed on the apical membrane of epithelial cells in the S1 and S2 segments of the proximal tubule, where it mediates most of the reabsorption of filtered glucose in concert with sodium. Pharmacological inhibition of this transporter reduces proximal glucose and sodium uptake, resulting in glucosuria, modest natriuresis, and a variable osmotic diuretic effect. Although these actions were initially viewed primarily in terms of their glycaemic and haemodynamic consequences, they are also relevant to stone disease because the proximal tubule substantially contributes to urinary volume, sodium balance, bicarbonate handling, urate transport, and the regulation of several lithogenic or inhibitory solutes.

The interaction between SGLT2 and proximal sodium–hydrogen exchange is particularly important. Experimental and physiological studies indicate functional coupling between SGLT2 activity, NHE3, and sodium–bicarbonate transport [8]. By modifying this transport system, SGLT2 inhibitors may influence urinary acid–base balance, although the resulting effect on urinary pH is not uniform. This is clinically relevant because urinary pH is a major determinant of uric acid crystallisation and affects calcium phosphate supersaturation [4,5]. Thus, changes in pH under SGLT2 inhibition should be interpreted in relation to baseline urinary phenotype and stone composition, rather than as a fixed class effect. SGLT2 inhibition also affects uric acid metabolism. These agents generally lower serum uric acid, largely through increased renal urate excretion, but the lithogenic meaning of uricosuria depends on urinary pH and relative supersaturation rather than urate excretion alone [4,5]. In parallel, short-term human studies have reported changes in urinary citrate and supersaturation profiles, suggesting that proximal tubular effects may extend beyond glucose and sodium transport [9]. Increased citrate excretion is potentially protective in calcium stone disease because citrate complexes calcium and inhibits crystal growth, whereas the net effect on uric acid stones remains more dependent on urinary pH [10,11].

### 3.2. Osmotic Diuresis, Natriuresis, and Urine Volume

By increasing glucose delivery beyond the proximal tubule, SGLT2 inhibitors generate an osmotic diuretic effect that may initially increase urine output and dilute lithogenic solutes. This mechanism is clinically attractive, since low urine volume remains one of the most consistent and modifiable risk factors for nephrolithiasis. Balasubramanian et al. reported that initiation of SGLT2 inhibitors may increase daily urine output by approximately 200–400 mL in patients with type 2 diabetes, a change that could reduce urinary concentrations of calcium, oxalate, and uric acid if fluid intake is maintained [12]. The natriuretic component appears less durable. After proximal sodium reabsorption is reduced, compensatory distal sodium handling limits sustained natriuresis, whereas glucosuria persists during active treatment. Dika et al. noted that this creates a clinically relevant balance: osmotic diuresis may lower supersaturation through dilution, but inadequate fluid replacement may produce the opposite effect by promoting volume contraction and concentrating stone-forming solutes [5]. This consideration is particularly important in older patients, individuals receiving loop or thiazide diuretics, patients with reduced thirst response, and those exposed to heat, fasting, acute illness, or perioperative fluid restriction.

In the SWEETSTONE trial, empagliflozin modified urinary citrate, pH, and relative supersaturation in non-diabetic calcium and uric acid stone formers, with reductions in calcium phosphate supersaturation in calcium stone formers and uric acid supersaturation in uric acid stone formers, while 24 h urine volume remained largely unchanged [4,9]. Taken together, these short-term human studies suggest that the urinary effects of empagliflozin may extend beyond simple dilution and may also involve changes in citrate, pH, and solute handling.

**Figure 1:**
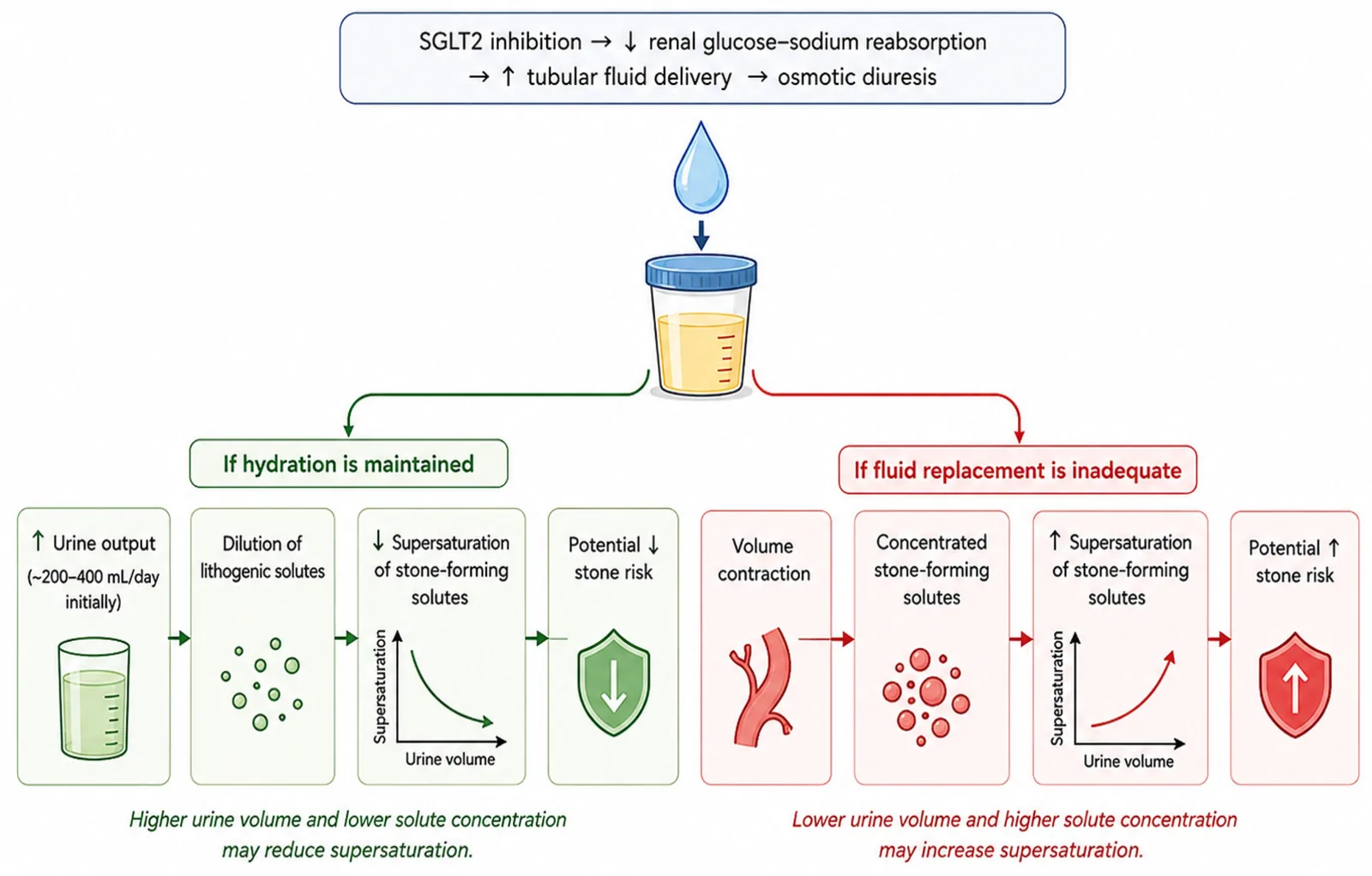
Hydration-dependent effects of SGLT2 inhibitor-associated osmotic diuresis on urinary concentration and stone risk.

In urological practice, the implications are straightforward. As shown in Figure 1, SGLT2 inhibitor-associated diuresis may lead to less concentrated urine, but this should not be assumed to confer reliable stone protection. Hydration advice remains necessary, and the treatment’s lithogenic effect should be interpreted alongside urinary pH, citrate, uric acid excretion, stone composition, and the patient’s risk of volume depletion.

### 3.3. Urinary pH, Ammonium Handling, and Acid–Base Balance

Urinary pH is central to stone formation because it influences the solubility of both uric acid and calcium phosphate. Low urinary pH favours uric acid crystallisation, even when urinary uric acid excretion is not markedly increased, whereas higher urinary pH may increase calcium phosphate supersaturation. This is particularly relevant in patients with obesity, insulin resistance, and type 2 diabetes, in whom impaired renal ammonium production contributes to persistently acidic urine [9]. SGLT2 inhibitors may influence acid–base handling through proximal tubular effects involving sodium–glucose transport, NHE3 activity, bicarbonate handling, glucosuria, and changes in substrate metabolism. However, the urinary pH response may vary depending on the baseline stone phenotype. In the SWEETSTONE trial, Anderegg et al. reported that empagliflozin modified urinary pH in non-diabetic calcium and uric acid stone formers, with corresponding changes in calcium phosphate and uric acid supersaturation according to stone phenotype [4]. These findings suggest that the urinary pH response to empagliflozin should not be regarded as a uniform class effect [13,14]. These findings suggest that the urinary pH response to empagliflozin may vary according to the studied population and baseline stone phenotype and should not yet be regarded as a uniform class effect. Their clinical meaning depends on baseline urinary pH, ammonium handling, stone composition, and associated changes in citrate and uric acid excretion. For urologists, urinary pH remains a key variable when assessing whether SGLT2 inhibition is likely to have favourable, neutral, or potentially unfavourable lithogenic consequences.

### 3.4. Citrate, Urate, Calcium, Phosphate, and Oxalate Handling

SGLT2 inhibition may affect several urinary solutes implicated in stone formation, but the strength of evidence varies across analytes. Citrate is the most consistently favourable signal. As an established inhibitor of calcium stone formation, citrate reduces free urinary calcium and interferes with crystal nucleation and growth. Ganesan et al. emphasised the central role of hypocitraturia in recurrent calcium nephrolithiasis and the rationale for alkali therapy in selected patients [14]. In this context, the increase in urinary citrate reported with empagliflozin by Anderegg et al. in SWEETSTONE and by Lombardi et al. in stone formers is clinically relevant, although it remains a surrogate endpoint rather than proof of recurrence prevention [4,15]. Urate requires a more cautious interpretation. Davies et al. showed that canagliflozin lowers serum uric acid and increases fractional uric acid excretion, supporting a renal uricosuric mechanism [15]. However, uric acid stone formation is driven primarily by urinary pH. McCormick et al. linked diabetes and gout to uric acid nephrolithiasis through persistently acidic urine, while SWEETSTONE suggested that higher uricosuria may coexist with lower uric acid supersaturation when pH and citrate shift favourably [13,14].

Calcium and phosphate handling appear more context-dependent. Balasubramanian et al. discussed possible increases in urinary calcium under SGLT2 inhibition, whereas Schietzel et al. proposed that distal tubular compensation, improved insulin sensitivity, and reduced hyperfiltration may attenuate this effect in some settings [9,12,14].

**Figure 2:**
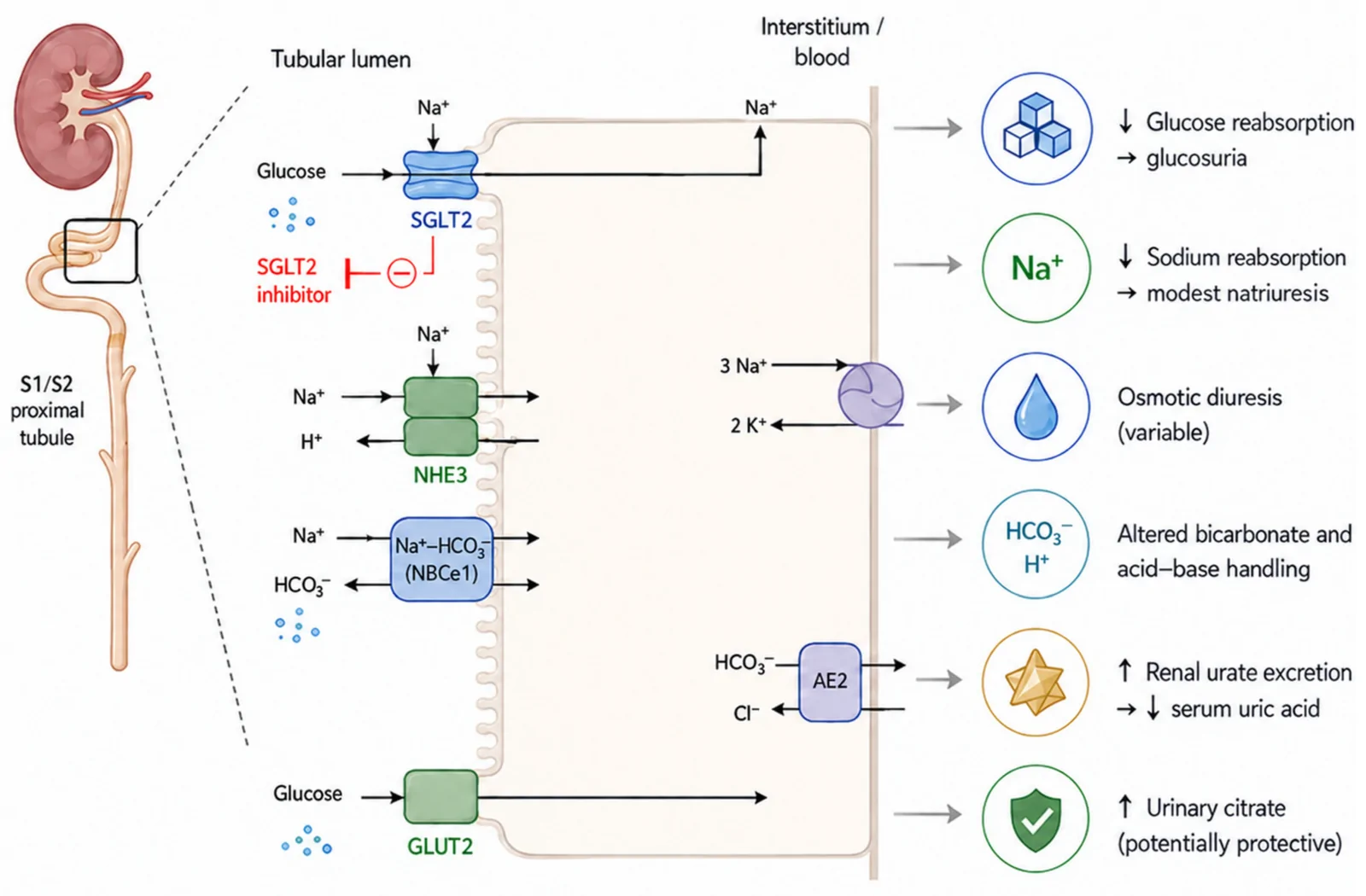
Renal tubular and urinary effects of SGLT2 inhibition relevant to kidney stone formation. SGLT2 inhibition reduces proximal glucose and sodium reabsorption and is associated with glucosuria, natriuresis, variable osmotic diuresis, changes in acid–base handling, increased renal urate excretion, and increased urinary citrate. SGLT2, sodium–glucose cotransporter 2; NHE3, sodium–hydrogen exchanger 3; NBCe1, sodium–bicarbonate cotransporter 1; AE2, anion exchanger 2; GLUT2, glucose transporter 2.

Oxalate remains the least defined component. Prezioso et al. and Ganesan et al. described dietary calcium, sodium, animal protein, and oxalate intake as important determinants of calcium oxalate stone risk in conventional stone prevention [14,16]. As shown in Figure 2, the available evidence suggests a potentially favourable effect on urinary citrate, whereas effects on urate, calcium, and phosphate appear more dependent on urinary pH, baseline phenotype, and study population. Data regarding urinary oxalate remain limited.

## 4. Effects on Urinary Chemistry and Stone-Specific Risk

### 4.1. Changes in 24 h Urinary Parameters

Twenty-four-hour urine testing remains the standard metabolic tool for identifying modifiable urinary risk factors in recurrent or high-risk stone formers. Prezioso et al. described its role in quantifying urine volume, pH, calcium, phosphate, urate, citrate, magnesium, sodium, potassium, sulphate, ammonium, and other analytes relevant to stone prevention [16]. In the context of SGLT2 inhibition, this assessment is useful because the expected effects extend beyond glucosuria and may involve several determinants of supersaturation. Individual changes in urinary pH, citrate, calcium, urate, or urine volume should be interpreted in the context of relative supersaturation, as favourable changes in a single parameter do not necessarily translate into reduced stone formation or recurrence. The most intuitive change is urine volume. Balasubramanian et al. reported that SGLT2 inhibitor initiation may increase daily urine output by approximately 200–400 mL, a modest effect that could contribute to solute dilution [12]. Kanbay et al. interpreted this volume effect as potentially relevant, but insufficient to explain the overall reduction in stone events observed across clinical datasets [3]. This view is supported by controlled studies in which changes in lithogenic markers occurred without major differences in 24 h urine volume.

Ganesan et al. emphasised the importance of hypocitraturia in calcium stone disease and the rationale for therapies that increase urinary citrate [14]. In healthy volunteers studied under standardised hydration conditions, short-term empagliflozin treatment increased urinary citrate by approximately 45%. Urinary pH decreased, whereas 24 h urine volume remained unchanged. Calcium phosphate supersaturation decreased and uric acid supersaturation increased, while calcium oxalate supersaturation was not significantly altered [4].

Other urinary parameters appear less reproducible. In a cross-sectional comparison of patients treated with SGLT2 inhibitors or GLP-1 receptor agonists, Harmacek et al. reported higher 24 h urine volume and urinary citrate in the SGLT2 inhibitor group, whereas urine pH, calcium, phosphorus, sulphate, oxalate, and uric acid did not differ significantly between groups [17]. Davies et al. showed that canagliflozin lowers serum uric acid and increases fractional uric acid excretion, but this uricosuric effect must be interpreted alongside urinary pH, since uric acid crystallisation is primarily pH-dependent [13,15]. Overall, available 24 h urine studies suggest a reproducible increase in citrate, modest or variable changes in urine volume, phenotype-dependent effects on pH, and complex changes in urate handling. For clinical interpretation, relative supersaturation is more informative than isolated analytes, because it integrates urine volume, pH, promoters, and inhibitors of crystallisation.

### 4.2. Effects on Calcium Oxalate Supersaturation

Calcium oxalate stones represent the most frequent form of nephrolithiasis, but the effect of SGLT2 inhibition on calcium oxalate supersaturation appears less consistent than for calcium phosphate or uric acid. From a urological standpoint, this distinction is important. A drug-induced increase in urinary citrate may be favourable, but calcium oxalate supersaturation also depends on urinary calcium, oxalate concentration, urine volume, and the balance between crystal inhibitors and promoters. Therefore, a favourable shift in one parameter cannot be assumed to translate into a lower risk of calcium oxalate crystallisation.

Available human data do not show a uniform reduction in calcium oxalate supersaturation. In healthy volunteers, empagliflozin increased urinary citrate but did not significantly reduce calcium oxalate supersaturation, while calcium phosphate supersaturation decreased, and uric acid supersaturation increased [4]. In stone formers enrolled in SWEETSTONE, the dominant biochemical effects were again seen in calcium phosphate and uric acid supersaturation rather than calcium oxalate. Ganesan et al. reported a substantial rise in urinary citrate, unchanged urinary oxalate, no significant change in urine volume, and a modest increase in urinary calcium among calcium stone formers treated with empagliflozin [14]. This pattern may explain why the calcium oxalate signal is less pronounced: the increase in citrate is potentially protective but may be partly offset by stable oxalate levels and changes in calcium excretion.

### 4.3. Effects on Calcium Phosphate Supersaturation

Short-term human studies have shown a more consistent effect on calcium phosphate supersaturation than on calcium oxalate supersaturation. In healthy volunteers, empagliflozin reduced calcium phosphate supersaturation, whereas in calcium stone formers enrolled in SWEETSTONE, a reduction of approximately 36% was observed after 2 weeks of treatment [4,14]. This biochemical profile is consistent with known determinants of calcium phosphate crystallisation. Higher urinary citrate may reduce free calcium activity, while a lower urinary pH reduces the tendency toward supersaturation of brushite and apatite. The observation is relevant because calcium phosphate stones, particularly brushite-containing stones, are often associated with a higher risk of recurrence, greater metabolic complexity, and a higher procedural burden.

The available evidence therefore supports a coherent short-term biochemical signal, but not a proven clinical effect. Whether reduced calcium phosphate supersaturation under SGLT2 inhibition translates into lower stone growth, fewer recurrent events, or reduced intervention rates remains unknown.

### 4.4. Effects on Uric Acid Supersaturation

Uric acid supersaturation is governed primarily by urinary pH, with urate excretion playing a secondary and context-dependent role. This distinction is essential in patients with diabetes, obesity, metabolic syndrome, or gout, in whom impaired ammonium buffering and persistently acidic urine favour uric acid crystallisation. McCormick et al. emphasised this mechanism in patients with gout and recurrent nephrolithiasis, where the metabolic milieu rather than urate load alone appears central to stone risk [13,18]. SGLT2 inhibitors complicate this profile by generally lowering serum uric acid while increasing renal urate excretion. Davies et al. demonstrated this uricosuric effect with canagliflozin, showing reduced serum uric acid and increased fractional uric acid excretion [15,19]. From a stone perspective, this could be unfavourable if urine remains strongly acidic. However, SWEETSTONE showed that empagliflozin reduced uric acid supersaturation by approximately 30% in uric acid stone formers, despite increased uricosuria, suggesting that changes in urinary pH and citrate may outweigh the higher urinary urate load in selected phenotypes [14]. The available data, therefore, support a phenotype-dependent interpretation. In patients with very acidic urine, the net effect of SGLT2 inhibition cannot be inferred from uricosuria alone.

Uric acid supersaturation, rather than urinary uric acid excretion, is the more relevant endpoint. Whether this biochemical improvement translates into fewer uric acid stones or lower recurrence rates remains to be established.

Table 1 summarises the expected effect of SGLT2 inhibition across the main stone phenotypes, together with the supporting biological rationale and the principal uncertainties in the current evidence.

## 5. Clinical Evidence on SGLT2 Inhibitors and Nephrolithiasis

Randomised data remain the most rigorous source of evidence for assessing whether the association between SGLT2 inhibition and lower stone risk may be causal. However, the available randomised evidence was not derived from trials primarily designed for nephrolithiasis. In large clinical programmes, urolithiasis was generally recorded as an adverse event, whereas smaller mechanistic trials assessed urinary chemistry and relative supersaturation over short follow-up periods. These two types of evidence are complementary but not interchangeable: adverse-event analyses provide a clinical signal, while mechanistic studies help explain how SGLT2 inhibition may alter the lithogenic urinary environment. The main randomised and randomised-derived studies relevant to stone disease are summarised in Table 2.

### 5.1. Evidence from Observational and Real-World Studies

Real-world studies have substantially expanded clinical evidence linking SGLT2 inhibitors with nephrolithiasis risk. Their value lies in large sample sizes, active-comparator designs, and the inclusion of patients with type 2 diabetes, gout, chronic kidney disease, obesity, and other comorbidities commonly encountered in urological practice. Across several cohorts, SGLT2 inhibitor use has been associated with fewer incidents or recurrent stone events compared with other glucose-lowering therapies, particularly DPP-4 inhibitors and GLP-1 receptor agonists.

Most studies define nephrolithiasis through diagnostic codes and capture healthcare-contact stone events rather than true stone formation, as shown in Table 3. Stone composition, urine pH, citrate, relative supersaturation, residual stone burden, hydration status, and dietary factors are usually unavailable. Therefore, observational data support a consistent clinical signal, but they cannot determine if the SGLT2 inhibitors prevent stone formation, reduce symptomatic presentation, or simply identify a lower-risk treated population.

### 5.2. Incident Nephrolithiasis Versus Stone Recurrence

The apparent reduction in nephrolithiasis reported with SGLT2 inhibitors depends strongly on how stone outcomes are defined. In pooled randomised data, urolithiasis was recorded as an adverse event, providing a useful clinical signal but not a stone-specific endpoint adjudicated by urologists [12]. In real-world datasets, nephrolithiasis is usually identified through diagnostic codes, which allows large-scale analyses but cannot reliably distinguish a first stone from a previously silent calculus, a recurrent symptomatic episode, or follow-up coding of known disease [21,22].

This distinction is relevant for urologists because prevention is judged mainly by recurrence rather than by the first appearance of a diagnostic code. Recurrent stone disease may reflect new stone formation, growth of residual fragments, migration of a known calculus, renal colic, obstruction, infection, or need for intervention. Kristensen et al. addressed this limitation more directly by analysing incident and recurrent nephrolithiasis separately in Danish registry cohorts and reported lower hazards for both outcomes among SGLT2 inhibitor initiators compared with active comparators [21]. McCormick et al. further strengthened the clinical relevance of this question by focusing on patients with pre-existing nephrolithiasis or gout, populations in whom recurrent events and uric acid stone risk are particularly important [13].

Table 4 places the main endpoints used in SGLT2 inhibitor studies in a urological context, distinguishing signals that are useful for hypothesis generation from those that would be needed to support stone-prevention practice.

### 5.3. Drug-Specific Versus Class-Wide Effects

Whether the reduction in nephrolithiasis observed with SGLT2 inhibitors reflects a molecule-specific effect or a broader class effect remains uncertain. The biological rationale is largely class-based: all approved SGLT2 inhibitors act on the proximal tubule, induce glucosuria, produce variable osmotic diuresis and natriuresis, and lower serum uric acid through increased renal urate excretion [25,26]. These shared mechanisms are relevant to stone biology, but they do not establish equivalent effects on urinary supersaturation, stone recurrence, or stone composition across individual agents.

Empagliflozin currently carries the strongest direct stone-specific evidence. It is the agent evaluated in the pooled randomised trial analysis showing fewer urolithiasis adverse events compared with placebo, and it is also the drug tested in the main human urinary supersaturation studies, including SWEETSTONE in non-diabetic calcium and uric acid stone formers [4,12]. For canagliflozin, the most relevant evidence concerns serum urate lowering and increased fractional uric acid excretion, whereas dapagliflozin has supportive experimental data in calcium oxalate models, particularly through effects on autophagy, oxidative stress, inflammation, and crystal deposition [15,24]. Most observational studies analyse SGLT2 inhibitors as a class and report fewer nephrolithiasis events compared with active glucose-lowering comparators [21,23]. However, these datasets generally lack sufficient granularity to compare individual agents by stone composition, urinary phenotype, dose, adherence, baseline stone burden, or recurrence mechanism. Therefore, no SGLT2 inhibitor can currently be selected solely for stone prevention. In urological practice, empagliflozin has the strongest direct evidence, whereas a class-wide anti-lithogenic effect remains plausible but unproven. Agent selection should continue to follow established cardiometabolic, renal, safety, and formulary indications, with stone phenotype guiding monitoring rather than drug choice.

## 6. Strengths, Limitations, and Remaining Uncertainties

### 6.1. Surrogate Urinary Outcomes Versus Clinical Stone Events

The evidence linking SGLT2 inhibitors to nephrolithiasis is strongest when biochemical and clinical findings are considered together, but these two categories of data should not be conflated. Twenty-four-hour urine parameters and relative supersaturation provide a rational mechanistic framework, particularly because they capture stone-type-specific changes in the urinary environment. This is why urinary supersaturation was selected as the principal endpoint in SWEETSTONE and why subsequent empagliflozin studies are important for understanding potential effects on calcium phosphate and uric acid risk profiles [4,14].

For urologists, however, favourable changes in citrate, pH, or relative supersaturation should be interpreted as risk modification rather than proof of prevention. Stone recurrence is influenced by factors not fully captured by urine chemistry, including residual fragments, papillary calcifications, Randall’s plaque, infection, obstruction, urinary stasis, stone burden, and thresholds for intervention. Conversely, clinical datasets showing fewer coded stone events provide population-level reassurance, but often lack stone composition, standardised imaging, and urinary phenotyping [12].

### 6.2. Confounding, Comparator Selection, and Outcome Misclassification

The observational evidence on SGLT2 inhibitors and nephrolithiasis is strengthened using active-comparator, new-user designs, propensity-score methods, high-dimensional adjustment, and target-trial emulation. These approaches reduce treatment-selection bias and make the findings more credible than simple comparisons with non-users [23,25]. Nevertheless, residual confounding remains difficult to exclude, particularly because variables central to stone prevention are rarely available in administrative datasets. 

The most important limitation for urological interpretation is the absence of stone phenotype. Stone composition is not a secondary descriptor in nephrolithiasis; it defines the underlying lithogenic pathway and directly informs metabolic evaluation, dietary counselling, pharmacological prevention, and follow-up strategy. Calcium oxalate, calcium phosphate, brushite, uric acid, struvite, and cystine stones differ substantially in pathogenesis and recurrence prevention [26,27]. Without composition data, it is not possible to determine whether the apparent benefit associated with SGLT2 inhibitors is driven preferentially by effects on uric acid, calcium phosphate, calcium oxalate, or mixed stone phenotypes. Other clinically relevant but usually unavailable variables include baseline stone burden, residual fragments, urinary pH, citrate excretion, dietary sodium, fluid intake, and adherence to preventive measures.

Comparator choice also influences interpretation. DPP-4 inhibitors and GLP-1 receptor agonists are preferable to untreated controls because they represent real therapeutic alternatives in patients with type 2 diabetes, but they are not neutral with respect to lithogenic risk. GLP-1 receptor agonists may alter body weight, appetite, gastrointestinal tolerance, and hydration patterns, whereas DPP-4 inhibitors have a less pronounced metabolic and renal profile. Therefore, the magnitude of risk reduction observed with SGLT2 inhibitors should be interpreted in relation to the comparator used rather than as an absolute estimate of anti-lithogenic efficacy. The consistency of findings across registry, claims-based, and TriNetX analyses is reassuring, but it does not eliminate comparator-specific bias [24].

Outcome misclassification adds a further layer of uncertainty. In randomised trials, urolithiasis events are generally captured as adverse events, whereas observational studies usually rely on diagnostic and, in some cases, procedure codes [16]. These definitions are suitable for large-scale signal detection, but they cannot provide the phenotypic resolution required for stone-prevention inference.

### 6.3. Heterogeneity in Populations, Exposure, and Follow-Up

The interpretation of SGLT2 inhibitor data is limited by heterogeneity in study populations, exposure definitions, and follow-up duration. Most clinical outcome studies were conducted in patients with type 2 diabetes and were structured around cardiometabolic treatment decisions rather than stone phenotype [21,22,28]. By contrast, SWEETSTONE enrolled non-diabetic stone formers with calcium or uric acid stones but assessed urinary supersaturation rather than clinical recurrence [14]. These populations address different questions and should not be treated as interchangeable. Exposure is also inconsistently defined. Some studies evaluate SGLT2 inhibitors as a class, whereas others are driven mainly by empagliflozin or do not provide drug-specific estimates. Treatment duration, adherence, discontinuation, switching, and concomitant use of diuretics, urate-lowering therapy, alkali treatment, or conventional stone-preventive medication are incompletely captured. This matters because urinary changes in volume, citrate, pH, and urate handling may appear early, whereas stone formation, stone growth, and symptomatic recurrence require longer observation. For urological practice, the consequence is important: the available evidence should not be extrapolated as a uniform effect across all stone formers. It is most applicable to metabolically complex patients, particularly those with diabetes, gout, obesity, or uric acid risk, while its relevance to non-diabetic recurrent calcium stone formers remains less certain. Future studies should prespecify population strata, drug exposure, treatment persistence, stone phenotype, and long-term follow-up sufficient to capture stone growth, symptomatic recurrence, and intervention.

## 7. What the Urologist Should Know

To avoid overinterpretation, the practical considerations below distinguish findings directly supported by the reviewed evidence from expert interpretation and from recommendations derived from established clinical guidelines.

### 7.1. Should SGLT2 Inhibitors Be Used for Stone Prevention?

SGLT2 inhibitors should not currently be initiated solely for kidney stone prevention. Their role in stone formers is best understood as adjunctive and context-dependent, particularly in patients who already have a conventional indication for treatment, such as type 2 diabetes, chronic kidney disease, heart failure, obesity, or metabolic syndrome. In these patients, nephrolithiasis may reasonably influence risk assessment and drug selection, but it does not constitute an independent indication for SGLT2 inhibitor therapy [29,30].

The rationale is strongest for metabolic stone formers, especially those at risk for uric acid stones, with low urinary pH, hypocitraturia, gout, or insulin-resistant phenotypes. These conditions overlap mechanistically with the cardiorenometabolic pathways targeted by SGLT2 inhibitors, including urate handling, tubular acid–base regulation, citrate excretion, inflammation, and renal metabolic stress [5]; however, this phenotype-specific relevance remains an expert interpretation based mainly on mechanistic and surrogate evidence rather than direct comparative clinical data.

This potential benefit should not displace established urological prevention. Fluid targets, dietary sodium reduction, adequate dietary calcium, oxalate and purine management, alkali therapy, thiazides, urate-lowering therapy, and follow-up imaging remain phenotype-directed interventions based on stone composition, recurrence history, and metabolic evaluation [16,31].

### 7.2. Should Nephrolithiasis Influence SGLT2 Inhibitor Selection?

The history of nephrolithiasis should not be considered a contraindication to SGLT2 inhibitor therapy when a standard cardiometabolic or renal indication is present. At the same time, it should not be ignored. In stone formers, SGLT2 inhibitor selection should remain primarily driven by approved indications, kidney function, cardiovascular status, tolerability, safety, and locally available guidance, rather than by an assumed stone-preventive effect [26,32].

From a urological perspective, nephrolithiasis becomes relevant mainly as a phenotyping and monitoring issue. Patients with uric acid stones, gout, low urinary pH, hypocitraturia, metabolic syndrome, or recurrent stones may be the group in whom SGLT2 inhibition is most biologically plausible, because these phenotypes overlap with urate handling, tubular acid–base regulation, citrate excretion, and renal metabolic stress [4,5]. However, these considerations support closer metabolic assessment rather than molecule selection solely based on stone history, and current evidence is insufficient to recommend SGLT2 inhibitor selection according to stone phenotype alone.

At present, empagliflozin has the strongest direct stone-specific evidence, including pooled randomised data and human urinary supersaturation studies. Evidence for other SGLT2 inhibitors is supported mainly by shared class mechanisms, urate-lowering effects, observational class-level analyses, and emerging mechanistic work [12,33,34].

### 7.3. Which Stone Phenotypes May Be Most Relevant?

The stone phenotypes most likely to be clinically relevant are those in which SGLT2 inhibition intersects with established metabolic drivers of lithogenesis. This applies particularly to uric acid stone formers and patients with persistently low urinary pH, gout, insulin resistance, obesity, or type 2 diabetes. In these patients, stone risk is often driven less by uric acid excretion itself than by impaired urinary buffering and acidic urine. The reduction in uric acid supersaturation observed in uric acid stone formers treated with empagliflozin, despite increased uricosuria, supports the importance of pH and citrate rather than isolated urate excretion [13,35]. For calcium oxalate stone formers, the evidence is more indirect. Human studies have not consistently shown a reduction in calcium oxalate supersaturation, but increased urinary citrate, and experimental data on reduced tubular injury, crystal adhesion, oxidative stress, inflammasome activation, osteopontin, and CD44 expression provide a plausible biological signal [36,37,38]. Based on the available mechanistic and surrogate data, recurrent calcium oxalate stone formers with hypocitraturia or cardiometabolic comorbidity may represent a subgroup of interest, although this remains an expert interpretation rather than an evidence-based treatment recommendation [37].

By contrast, there is no convincing rationale to prioritise SGLT2 inhibitors for infection stones, cystine stones, or anatomically driven stone disease. In these settings, stone prevention remains dominated by infection control, urinary drainage, cystine-specific therapy, anatomical correction, and conventional metabolic management. Overall, SGLT2 inhibitors are most plausibly relevant in metabolic stone phenotypes—particularly uric acid risk, calcium phosphate risk, and recurrent calcium oxalate disease with hypocitraturia or cardiometabolic comorbidity—rather than across nephrolithiasis as a uniform category; however, this interpretation is based largely on mechanistic and short-term biochemical evidence and should not be regarded as a phenotype-specific treatment recommendation.

### 7.4. Hydration, Diuretics, Diet, and Metabolic Monitoring

The osmotic diuresis associated with SGLT2 inhibition has practical relevance in stone formers, but it should not be overinterpreted as a protective intervention by itself. A modest increase in urine output may reduce solute concentration, yet this effect depends on maintained fluid intake and may be offset by volume depletion, intercurrent illness, heat exposure, fasting, or concomitant diuretic therapy [5,12].

Frailty is common among older patients with diabetes, chronic kidney disease, or heart failure and may complicate therapeutic decisions because of concerns regarding treatment tolerability. However, analyses of DAPA-HF and DELIVER found consistent reductions in worsening heart failure or cardiovascular death across frailty categories, while DAPA-CKD showed preserved kidney and cardiovascular benefits with no meaningful variation in safety; taken together, these findings suggest that frailty alone should not exclude otherwise appropriate SGLT2 inhibitor therapy [38,39].

Concomitant diuretic use deserves specific review. Thiazides may remain indicated for hypercalciuric calcium stone disease, whereas loop diuretics may increase urinary calcium and can aggravate volume instability in susceptible patients. When SGLT2 inhibitors are combined with diuretics or renin–angiotensin system blockers, clinical monitoring should focus on hydration status, orthostatic symptoms, renal function, and changes in urinary concentration rather than on stone risk alone [26].

SGLT2 inhibitors do not replace sodium restriction, adequate dietary calcium, oxalate management, moderation of animal protein or purine intake when appropriate, alkali therapy, thiazides, or urate-lowering therapy. In patients with recurrent stones or complex metabolic profiles, 24 h urine testing remains useful before and after major therapeutic changes, with particular attention to urine volume, pH, citrate, calcium, oxalate, uric acid, sodium, phosphate, and relative supersaturation [15,16].

### 7.5. Infection-Related and Perioperative Considerations

Infection-related safety is relevant in stone practice because obstruction, residual fragments, stents, nephrostomy tubes, and infected stones may increase the clinical consequences of urinary infection. Genital mycotic infections are the most consistent genitourinary adverse effect of SGLT2 inhibitors, whereas severe urinary tract infections do not appear to increase uniformly across major datasets. Nevertheless, recurrent UTI, infected obstruction, struvite stones, indwelling drainage, or planned endourological intervention should prompt individualised assessment rather than automatic discontinuation or uncritical continuation [26,32].

Perioperative management is particularly important for FURS, PCNL, and procedures requiring anaesthesia or prolonged fasting. Reduced oral intake, perioperative fluid shifts, infection, and acute illness may increase the risk of dehydration and, rarely, euglycemic ketoacidosis in susceptible patients. For this reason, perioperative management of SGLT2 inhibitors should follow institutional or international guidance and be coordinated with the prescribing clinician, as these recommendations are based on general perioperative safety guidance rather than nephrolithiasis-specific studies. From a urological standpoint, infection control, adequate hydration, and postoperative clinical stability remain standard perioperative principles, while the timing of SGLT2 inhibitor interruption and reintroduction should follow established prescribing and perioperative guidance.

### 7.6. Multidisciplinary Management of the Metabolic Stone Former

The potential relevance of SGLT2 inhibitors reinforces the need to manage recurrent nephrolithiasis as a metabolic disease rather than as an isolated surgical problem. The urologist defines stone composition, recurrence pattern, residual stone burden, infection risk, and urinary phenotype. These data are essential because the same drug exposure may have different implications in uric acid, calcium phosphate, calcium oxalate, infection-related, or cystine stone disease.

**Figure 3:**
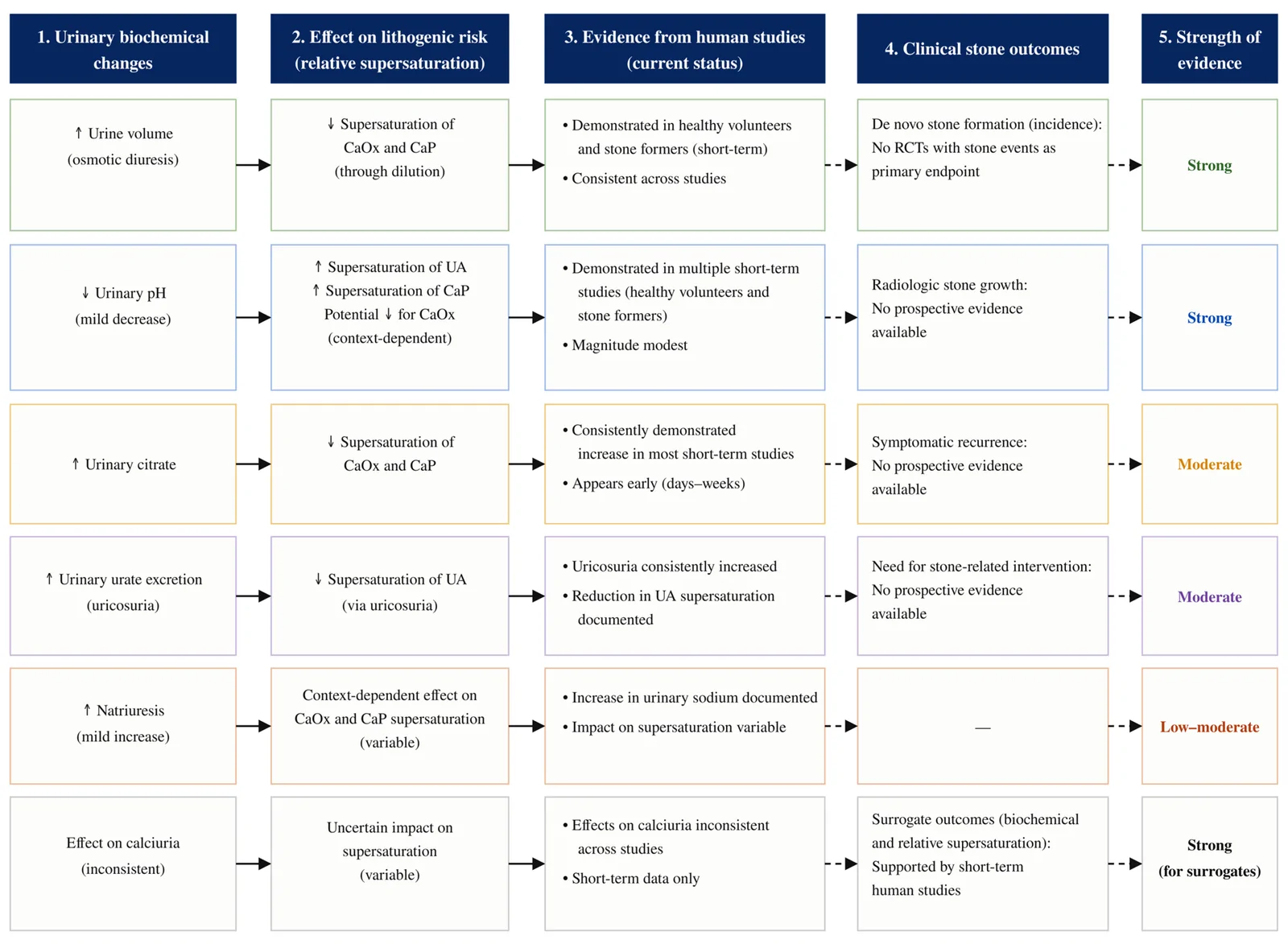
Evidence pathway linking SGLT2 inhibitor-associated changes in urinary biochemistry to relative supersaturation and clinical stone outcomes. Solid arrows indicate evidence-supported biochemical relationships, whereas dashed arrows highlight the current evidence gap between surrogate changes and clinical outcomes. Evidence synthesis based on clinical and review data concerning urinary biochemical effects and stone outcomes [4,11,13,14,15,16,17,20,21,22]. CaOx, calcium oxalate; CaP, calcium phosphate; UA, uric acid; RCTs, randomized controlled trials.

Therapeutic decisions regarding SGLT2 inhibitors should remain shared with the clinician responsible for diabetes, kidney disease, heart failure, or cardiovascular risk. Nephrologists and diabetologists can assess kidney-function thresholds, cardiorenal indications, drug interactions, and metabolic safety; cardiologists may be central when heart failure or vascular disease drives treatment; dietitians are important when stone-directed dietary advice must be reconciled with diabetes, CKD, obesity, or cardiovascular recommendations. In this model, SGLT2 inhibitors are not stand-alone stone-preventive drugs. Their most appropriate role is as part of a broader, phenotype-directed strategy for metabolically complex stone formers, particularly when standard treatment indications already exist, as shown in Figure 3 [5,29].

## 8. Future Directions

Future studies should move beyond coded nephrolithiasis events and evaluate whether SGLT2 inhibitors reduce new stone formation, stone growth, symptomatic recurrence, or the need for intervention. Dedicated prospective trials should include baseline stone composition and burden, residual fragments, relevant comorbidities, concomitant preventive treatment, serial 24 h urine testing, and standardised imaging.

Nephrolithiasis should not be treated as a single outcome. Composition-specific analyses are needed, particularly in uric acid and calcium phosphate stone formers, where current mechanistic and supersaturation data are most suggestive. Urinary pH, citrate, urate handling, and relative supersaturation should be assessed as potential mediators, while clinical endpoints such as radiological stone growth, recurrent symptoms, obstruction, infection, and stone-related procedures should be reported separately.

## 9. Conclusions

SGLT2 inhibitors are increasingly relevant to urological practice because many patients with nephrolithiasis also have type 2 diabetes, chronic kidney disease, heart failure, gout, or other cardiometabolic comorbidities. Current evidence suggests that SGLT2 inhibition may favourably modify selected determinants of lithogenesis, particularly urinary citrate and stone-specific supersaturation profiles. SGLT2 inhibitors should therefore not be initiated solely for kidney stone prevention, but could be used in patients with an established cardiovascular, renal, or metabolic indication, particularly in selected metabolically complex stone formers. Dedicated, long-term randomised studies incorporating stone composition, standardised imaging, 24 h urinary profiles, recurrence, and stone-related interventions are required before these agents can be incorporated into formal nephrolithiasis-prevention strategies.

## Figures and Tables

**Table 1:** Phenotype-specific interpretation of the potential effects of SGLT2 inhibition on nephrolithiasis.

Stone Phenotype	Expected Net Effect	Phenotype-Specific Interpretation	Evidence and Main Uncertainty
Uric acid stones	Potentially favourable in selected patients	Changes in urinary pH and citrate may reduce uric acid supersaturation despite increased renal urate excretion.	Direct but limited short-term evidence [17]. The effect may be unfavourable or neutral if urine remains markedly acidic; recurrence data are lacking.
Calcium phosphate-containing stones	Potentially favourable	Increased urinary citrate and a modest reduction in urinary pH may lower calcium phosphate supersaturation.	Consistent short-term biochemical evidence [9,17]. Brushite-specific and recurrence data are unavailable.
Calcium oxalate stones	Uncertain	Increased citrate may be favourable, but calcium oxalate supersaturation has not consistently decreased.	Limited biochemical evidence [9,17,19]. The net effect on stone recurrence remains unclear.
Mixed calcium stones	Probably phenotype-dependent	The net effect may depend on the relative contributions of calcium oxalate and calcium phosphate and on baseline urinary chemistry.	Indirect inference from mechanistic studies [9,17]. Mixed stone composition has not been adequately evaluated.
Infection stones	No established effect	SGLT2 inhibition does not target the principal drivers of infection stone formation.	No stone-specific evidence. Findings from metabolic stone phenotypes should not be extrapolated.
Cystine stones	No established effect	No direct effect on cystine excretion or cystine supersaturation has been demonstrated.	No stone-specific evidence. Any benefit related to urine volume remains speculative.

**Table 2:** Randomised and randomised-derived evidence on SGLT2 inhibitors and kidney stone risk.

Study	Design and Population	Intervention and Comparator	Treatment Duration	Outcome Definition	Main Findings	Outcome Ascertainment and Methodological Appraisal
Balasubramanian et al. [12].	Pooled analysis of 20 placebo-controlled phase I–IV empagliflozin trials, including EMPA-REG OUTCOME; 15,081 patients with type 2 diabetes	Empagliflozin 10 or 25 mg vs. placebo	Median treatment exposure: 549 days with empagliflozin and 543 days with placebo	Incident urinary tract stone events identified using a predefined MedDRA collection	0.63 vs. 1.01 events/100 patient-years; IRR 0.64 (95% CI 0.48–0.86). Sensitivity analysis: IRR 0.62 (95% CI 0.45–0.85)	Stone events were identified retrospectively from adverse-event reporting and were not a prespecified primary nephrolithiasis endpoint. No systematic imaging, stone-composition assessment, or recurrence adjudication was performed.
Cosentino et al. [20]	Meta-analysis of 27 randomised trials in patients with type 2 diabetes; 32,931 participants overall; 16 trials reported ≥1 nephrolithiasis event	SGLT2 inhibitors vs. placebo or active comparators	Included trials had a duration of ≥52 weeks	Nephrolithiasis reported by trial investigators as serious adverse events	62 events in SGLT2 inhibitor groups vs. 44 in controls; OR 0.85 (95% CI 0.57–1.26)	Nephrolithiasis was not a prespecified outcome in the underlying trials. Event numbers were low, and ascertainment depended on trial safety reporting rather than dedicated stone assessment.
Anderegg et al.—SWEETSTONE trial [4]	Randomised, double-blind, placebo-controlled, single-centre crossover phase II trial including 53 non-diabetic adults with previous kidney stone events: 28 calcium stone formers and 25 uric acid stone formers	Empagliflozin 25 mg/day vs. placebo	Two weeks per treatment period in crossover design	Prespecified primary outcomes: relative supersaturation ratios for CaOx, CaP, and uric acid	Calcium stone formers: CaP RSR −36% (95% CI −48% to −21%; *p* < 0.001); CaOx RSR −1% (95% CI −15% to 16%). Uric acid stone formers: UA RSR −30% (95% CI −44% to −12%; *p* = 0.002)	Biochemical surrogate endpoints were prespecified and prospectively assessed. The trial was not designed to evaluate clinical stone recurrence, stone growth, or need for urological intervention.

Abbreviations: BMI, body mass index; CI, confidence interval; DPP-4, dipeptidyl peptidase-4; eGFR, estimated glomerular filtration rate; GLP-1, glucagon-like peptide-1; HbA1c, glycated haemoglobin; HR, hazard ratio; ICD, International Classification of Diseases; SGLT2, sodium–glucose cotransporter 2.

**Table 3:** Observational and real-world evidence on SGLT2 inhibitors and nephrolithiasis.

Study	Design and Population	Comparator	Follow-Up	Outcome Definition	Adjustment Strategy	Effect Estimate	Outcome Ascertainment/Methodological Appraisal
Kristensen et al., 2021 [21]	Danish nationwide active-comparator, new-user cohort. Main analysis: 12,325 propensity-score-matched pairs without previous nephrolithiasis; recurrence analysis: 731 matched pairs with previous nephrolithiasis	SGLT2i vs. GLP-1RA; DPP-4i used in sensitivity analysis	Median 2.1 years for SGLT2i and 1.9 years for GLP-1RA after matching	Primary: incident kidney/ureter stone diagnosed in inpatient or outpatient registry data. Supplementary: recurrent nephrolithiasis in patients with prior stone disease	Incident and recurrent nephrolithiasis identified from registry diagnosis codes	Incident NL vs. GLP-1RA: HR 0.51 (95% CI 0.37–0.71). Recurrent NL: HR 0.68 (95% CI 0.48–0.97). Sensitivity comparison vs. DPP-4i: HR 0.61 (95% CI 0.41–0.88)	Incident nephrolithiasis was the prespecified primary outcome; recurrence was a supplementary analysis. Outcomes were derived from Danish registry diagnosis codes and were not adjudicated by chart review. Stone composition and urinary phenotype were unavailable.
Yeh et al., 2025 [22]	TriNetX cohort of adults with T2D initiating glucose-lowering therapy; 250,000 matched pairs for SGLT2i vs. DPP-4i and 241,142 matched pairs for SGLT2i vs. GLP-1RA	SGLT2i vs. DPP-4i and SGLT2i vs. GLP-1RA	Up to 5 years	Incident nephrolithiasis recorded in the TriNetX electronic health-record network	1:1 propensity-score matching using demographics, comorbidities, baseline medications and relevant laboratory variables; post-matching covariate balance assessed by standardised differences	SGLT2 inhibitor vs. DPP-4i: HR 0.86 (95% CI 0.83–0.90); vs. GLP-1RA: HR 0.90 (95% CI 0.86–0.94)	Nephrolithiasis was the clinical outcome of interest, but ascertainment was based on routinely collected EHR/diagnostic data rather than stone-specific adjudication. No information on stone composition, imaging phenotype or urinary chemistry was available.
Chung et al. [23]	Multi-database cohort using Taiwan NHIRD and TriNetX. After PSM: 112,701 SGLT2i/DPP-4i pairs in NHIRD and 114,052 pairs in TriNetX; patients with previous urolithiasis were excluded	SGLT2i vs. DPP-4i	From treatment initiation until outcome, death, or end of study; median time to urolithiasis: 1.91 years in NHIRD and 3.35 years in TriNetX	Primary: incident urolithiasis identified using ICD-9/10 codes. Additional clinical outcome: urolithiasis requiring surgery, defined by a stone diagnosis plus a procedure code during the same encounter	1:1 propensity-score matching. Fully adjusted Cox models included age, sex, diabetes duration, race where available, comorbidities and concomitant medications	NHIRD: incident UL HR 0.82 (95% CI 0.77–0.87); surgery HR 0.72 (95% CI 0.63–0.82). TriNetX: incident UL HR 0.84 (95% CI 0.78–0.90); surgery HR 0.71 (95% CI 0.56–0.89)	Outcomes were explicitly defined using administrative diagnostic and procedure codes; no individual clinical adjudication was performed. Importantly, incident urolithiasis and stone-related surgery were analysed separately. No stone composition or urinary biochemical data were available.
Liu et al., 2025 [24]	Multinational TriNetX retrospective cohort of adults with diabetes. After PSM: 358,096 matched pairs for SGLT2i vs. DPP-4i and 371,374 pairs for SGLT2i vs. GLP-1RA	SGLT2i vs. DPP-4i and SGLT2i vs. GLP-1RA	1 year after index prescription	Primary outcome: incident nephrolithiasis, defined using ICD-10 N20–N23 codes	Greedy nearest-neighbour propensity-score matching; covariates included age and recorded metabolic, cardiovascular and other baseline comorbidities listed in the study; subgroup analyses by HbA1c, age, BMI and eGFR	SGLT2i vs. DPP-4i: HR 0.825 (95% CI 0.795–0.855); vs. GLP-1RA: HR 0.812 (95% CI 0.784–0.840)	Incident nephrolithiasis was the specified primary outcome and was derived from ICD coding rather than adjudicated stone events. Follow-up was limited to 1 year, and stone composition, imaging characteristics and urinary phenotype were unavailable.

Abbreviations: BMI, body mass index; CI, confidence interval; DPP-4, dipeptidyl peptidase-4; eGFR, estimated glomerular filtration rate; GLP-1, glucagon-like peptide-1; HbA1c, glycated haemoglobin; HR, hazard ratio; ICD, International Classification of Diseases; SGLT2, sodium–glucose cotransporter 2.

**Table 4:** Urological relevance of stone outcomes in SGLT2 inhibitor studies.

Endpoint Used in SGLT2 Studies	Clinical Information Provided	Limitation for Stone-Prevention Inference
Incident nephrolithiasis in diabetes cohorts	Indicates fewer newly recorded stone events among SGLT2 inhibitor users compared with other glucose-lowering therapies, as reported in registry and TriNetX-based analyses [21,23,25]	May include previously silent stones or first documented presentations rather than true de novo stone formation
Recurrent nephrolithiasis in prior stone formers	Provides the most relevant clinical signal for prevention when assessed in active-comparator, new-user designs [13,21]	May combine new stone formation, residual fragment growth, stone migration, or repeated coding of known disease
Urolithiasis adverse events in randomised trials	Offers a randomised clinical signal with less treatment-selection bias than observational datasets [12]	Usually lacks imaging confirmation, stone composition, recurrence adjudication, and urinary phenotype
ICD-coded kidney or ureteric stones	Enables large-scale assessment of healthcare-contact stone disease in routine practice [23]	Does not define stone size, laterality, burden, composition, or lithogenic mechanism
Stone-related procedures	Identifies clinically significant disease requiring intervention and captures part of the healthcare burden [23]	Influenced by local treatment thresholds, access to endourology, symptoms, anatomy, stone size, and residual fragments
Relative supersaturation endpoints	Links SGLT2-related urinary changes to stone-type-specific crystallisation risk [4]	Remains a surrogate outcome and cannot establish clinical recurrence prevention without longitudinal stone endpoints

## Data Availability

The data presented in this study are available on request from the corresponding author due to institutional approval and applicable patient confidentiality requirements.

## Abbreviations

The following abbreviations are used in this manuscript:

AE2	Anion exchanger 2
BMI	Body mass index
CI	Confidence interval
CKD	Chronic kidney disease
DPP-4	Dipeptidyl peptidase-4
eGFR	Estimated glomerular filtration rate
GLP-1	Glucagon-like peptide-1
GLUT2	Glucose transporter 2
HbA1c	Glycated haemoglobin
HR	Hazard ratio
ICD	International Classification of Diseases
ICD-9	International Classification of Diseases, Ninth Revision
ICD-10	International Classification of Diseases, Tenth Revision
IRR	Incidence rate ratio
MedDRA	Medical Dictionary for Regulatory Activities
NHE3	Sodium–hydrogen exchanger 3
OR	Odds ratio
SGLT2	Sodium–glucose cotransporter 2
T2DM	Type 2 diabetes mellitus
